# Updates in Anticoagulation Therapy Monitoring

**DOI:** 10.3390/biomedicines9030262

**Published:** 2021-03-06

**Authors:** Hannah L. McRae, Leah Militello, Majed A. Refaai

**Affiliations:** Department of Pathology and Laboratory Medicine, Transfusion Medicine Division, Hemostasis and Thrombosis Unit, University of Rochester Medical Center, Rochester, NY 14642, USA; hannah_mcrae@urmc.rochester.edu (H.L.M.); leah_militello@urmc.rochester.edu (L.M.)

**Keywords:** anticoagulant, anticoagulation, warfarin, direct oral anticoagulant, heparin, low molecular weight heparin, direct thrombin inhibitor, point of care testing, anticoagulation monitoring, coagulation

## Abstract

In the past six decades, heparin and warfarin were the primary anticoagulants prescribed for treatment and prophylaxis of venous thromboembolism worldwide. This has been accompanied by extensive clinical knowledge regarding dosing, monitoring, and reversal of these anticoagulants, and the resources required to do so have largely been readily available at small and large centers alike. However, with the advent of newer oral and parenteral anticoagulants such as low molecular weight heparins, factor Xa inhibitors, and direct thrombin inhibitors in recent years, new corresponding practice guidelines have also emerged. A notable shift in the need for monitoring and reversal agents has evolved as well. While this has perhaps streamlined the process for physicians and is often desirable for patients, it has also left a knowledge and resource gap in clinical scenarios for which urgent reversal and monitoring is necessary. An overview of the currently available anticoagulants with a focus on the guidelines and available tests for anticoagulant monitoring will be discussed in this article.

## 1. Introduction

Since the initial discovery of heparin in 1916, there have been countless clinical and scientific advances in the pharmacophysiology of anticoagulation. This started from the commercial production and first clinical trials involving heparin in the 1930s, to the discovery of coumarin in the 1940s and the subsequent development of warfarin as a rodenticide in 1948. This was then followed by decades-long widespread clinical use of heparin and warfarin and the more recent development of new, targeted oral anticoagulants in the past 10–15 years [1,2,3]. Anticoagulation is indicated in a broad range of clinical scenarios, including (but not limited to) the management of venous and/or arterial thromboembolism, treatment of disseminated intravascular coagulation, the flushing of lines such as in hemodialysis, cardiopulmonary bypass, or extracorporeal membrane oxygenation (ECMO). Anticoagulation can also be used prophylactically in patients with atrial fibrillation, artificial valves, and in the post-operative and critical care settings [4].

Anticoagulants are often categorized according to the mechanism of action (Table 1). Heparins are subcategorized as unfractionated heparin (UFH) (given intravenously or subcutaneously) and low molecular weight heparins (LMWH) including enoxaparin (Lovenox**^®^**/Clexane^®^, Sanofi, Paris, France), dalteparin (Fragmin**^®^**, Pfizer, New York, NY, USA), tinzaparin (Innohep**^®^**, LEO Pharma, Ballerup, Denmark) and nadroparin (Fraxiparine**^®^**/Fraxodi**^®^**, Aspen Pharmacare, Durban, South Africa), which are administered subcutaneously. Warfarin, acenocoumarol, and phenprocoumon are known as vitamin K antagonists, and have historically been the most ubiquitous oral anticoagulants prescribed for long-term anticoagulation. Factor Xa inhibitors consist of fondaparinux (Atrixa**^®^**, Aspen Pharmacare, Durban, South Africa)) (a synthetic anticoagulant), which is administered subcutaneously, as well as the direct oral anticoagulants (DOAC) rivaroxaban (Xarelto**^®^**, Janssen Pharmaceuticals, Beerse, Belgium), apixaban (Eliquis**^®^**, Bristol-Myers Squibb, New York, NY, USA), edoxaban (Savaysa**^®^**, Daiichi-Sankyo, Tokyo, Japan), and betrixaban (Bevyxxa**^®^**, Portola, South San Francisco, CA, USA). The final class of anticoagulants is the direct thrombin inhibitors (DTI), which include dabigatran (Pradaxa**^®^**, Boehringer Ingelheim, Ingelheim am Rhein, Germany) (a DOAC), and the parenterally administered bivalirudin (Angiomax**^®^**, Sandoz, Holzkirchen, Germany) and argatroban (Acova**^®^**, Texas Biotechnology Corporation, Houston, TX, USA).

In addition to the logistics associated with the different types of administration of anticoagulation, the clinical indication for each anticoagulant varies due to discrepancies in the risk of adverse drug events (particularly thrombotic vs. hemorrhagic risk), therapeutic index, mechanism of drug clearance (i.e., hepatic vs. renal), drug half-life, requirements for therapeutic monitoring, and potential and mechanism of anticoagulant reversal [4,5]. This article provides an overview of the currently available anticoagulants, with a primary focus on the guidelines and available tests for therapeutic anticoagulation monitoring.

## 2. Vitamin K Antagonists

Vitamin K antagonists (VKA) are oral anticoagulants that inhibit the vitamin K epoxy reductase enzyme, which is required for the conversion of vitamin K to its active form, vitamin KH2. The vitamin K-dependent coagulation factors (II, VII, IX, and X) depend on vitamin KH2 to become synthesized by the liver [6]. Warfarin is the most common VKA used clinically in the United States, while others such as acenocoumarol and phenprocoumon are frequently used in other countries. VKA are the most commonly prescribed oral anticoagulants worldwide, though fewer patients are being prescribed VKA now as more Xa- and IIa-inhibiting direct oral anticoagulants have become increasingly prevalent in the past decade [7]. VKA are clinically indicated in the treatment and prophylaxis of venous thromboembolism (VTE) and pulmonary embolism, and in the setting of heart failure, atrial fibrillation, acute coronary syndrome, prosthetic heart valve, stroke, and antiphospholipid syndrome [8,9]. Contraindications include bleeding diathesis, thrombocytopenia, central nervous system tumors, major trauma, uncontrolled hypertension, active bleeding, and pregnancy, as VKA cross the placenta and may induce fetal hemorrhage as well as increases the risk for bleeding complications during delivery [9,10].

One of the main advantages to VKA therapy is the body of research and evidence-based practice guidelines that stem from decades of use worldwide. As a result, there is a high degree of clinical familiarity with the drug. In addition, VKA are cheap and easily accessible compared to DOACs; a 2018 study in Britain revealed that DOACs are prescribed to 31% of patients treated for atrial fibrillation, but account for approximately 93% of National Health Service (NHS) expenditure on anticoagulants (referring to prescription costs only) [11]. VKA have also been shown to be safer and more efficacious than other oral anticoagulants in patients with certain conditions, such as prosthetic heart valves and recurrent thrombosis in the setting of antiphospholipid syndrome [12,13]. VKA are also quickly and easily reversed, which is necessitated by many scenarios from planned surgeries to major trauma and intracranial hemorrhage. Depending on the urgency and extent of international normalized ratio (INR) correction required, reversal can be achieved by VKA discontinuation (or abruption), the administration of oral or IV vitamin K, transfusion of fresh frozen plasma (FFP), and replacement of vitamin K-dependent coagulation factors via infusion of prothrombin complex concentrates.

However, there are several adverse effects associated with VKA therapy that make DOAC a better option in some cases, including high rates of serious bleeding complications. According to the results of 33 meta-analyses, the rate of major VKA-related bleeding events is 7.2 per 100 patient-years, and fatal bleeds occur at a rate of 1.3 per 100 patient-years [14]. VKA are also shown to be unpredictable and associated with high rates of thromboembolic and bleeding complications in patients with atrial fibrillation; a study of 6454 patients with atrial fibrillation revealed that patients were outside the therapeutic range almost 50% of the time, thus increasing the risk for either thrombosis (below the range) or bleeding (above the range) [15]. Furthermore, VKA require frequent monitoring, and diet and co-medications can have considerable implications in patients taking VKA, either enhancing or inhibiting their anticoagulant effect [9].

VKA also have a delayed onset of action as compared to other anticoagulants, typically requiring 24–72 h, and approximately 5–7 days to reach their peak therapeutic effect after initiation. The half-life is approximately 40 h on average (ranging from 20–60 h); however, there is great variability in half-life duration between patients [8]. The therapeutic level of VKA is measured by prothrombin time (PT) and INR, which is a standardized ratio developed by the World Health Organization (WHO) in the 1980s specifically for VKA monitoring, as PT varies greatly between laboratories [16]. WHO developed its procedure to mitigate the discrepancy in tissue factor (TF) activity between PT reagents and thus allow for the expression of PT results on a common scale, i.e., INR. The INR is derived from the International Sensitivity Index (ISI), which was developed by WHO and quantifies the reactivity of individual PT reagents and analyzers. In addition, each center has its own geometric mean PT (MNPT), which is the average PT calculated from a least 20 normal donors of both sexes, tested on the same local analyzer and under the same test conditions as the patient PT. The formula for INR is INR = (patient’s PT/MNPT)^ISI^ [17,18].

In order to properly assess INR after initiation of VKA therapy, the baseline PT, activated partial thromboplastin times (aPTT), and INR values are obtained; this ensures proper calculation of the therapeutic target, and is especially important in patients with naturally prolonged PT and/or aPTT, such as in the case of antiphospholipid syndrome. In addition, it is advisable to assess the patient’s liver function to identify potential issues with the metabolism of VKA or baseline hemostatic issues [9]. VKA have a narrow therapeutic index (typically an INR of 2.0–3.0, though this may be higher in the setting of artificial heart valves or conditions such as antiphospholipid syndrome), and the dosages required to achieve this range are inconsistent from patient to patient. The correlation between dose and anticoagulant response can be affected by genetic factors as well as environmental variables, such as dietary and nutritional intake, drug interactions, illness and injury, etc., all of which affect the absorption and pharmacokinetics of VKA and vitamin K [5]. Depending on fluctuations in INR, clinicians may either adjust the patient’s warfarin dosing accordingly, or instruct them to alter their dietary habits.

**Figure 1:**
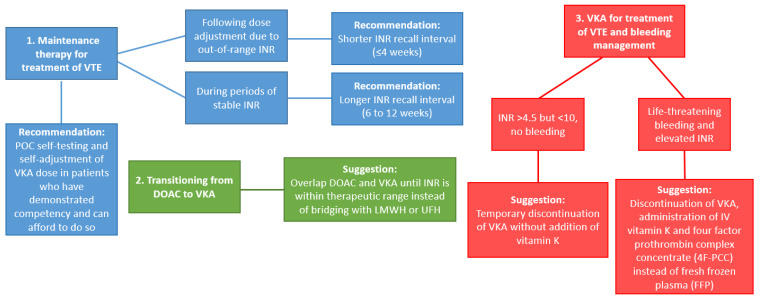
Updates to recommendations for VKA management (Based on ASH guidelines [22]). Abbreviations: VTE: venous thromboembolism; POC: point-of-care; VKA: Vitamin K antagonists; INR: international normalized ratio; DOAC: Direct oral anticoagulant; LMWH: low molecular weight heparins; UFH: unfractionated heparin.

Careful monitoring via INR is typically recommended at the initiation of VKA therapy, and is usually performed daily in hospitalized patients, and one to three times per week in outpatients until the dose is properly adjusted. Monitoring may be more frequent in patients for whom there is difficulty achieving INR within the therapeutic range, and may eventually decrease to intervals between every two to four weeks once the INR has stabilized for at least one week [9]. Traditionally VKA monitoring has required patients to travel to an outpatient laboratory or clinic and undergo a venous blood draw for each INR; however, in the past 10–15 years several point-of-care (POC) devices have been developed and approved for both clinical and home use. This allows patients to be tested either at their regular clinic appointments (thus alleviating the need for extra travel), or at their convenience at home using a personal INR meter, from which the results are uploaded to their electronic medical records for direct supervision by their medical provider. Perhaps unsurprisingly, patient self-testing has been associated with improved quality of life and cost savings as compared to traditional INR monitoring [19]. However, recent studies have shown that POC INR devices are associated with decreased precision when the INR is greater than 3.0, and general unreliability once the INR exceeds 4.0 [20,21]. These devices are therefore best suited to patients who are compliant with their diet and VKA therapy, and who maintain stable INRs within the therapeutic range and with little variability long-term. As new data emerges, the American Society of Hematology (ASH) guidelines for best practices in VKA monitoring and reversal continue to be updated (Figure 1) [22].

## 3. Heparin

Heparin is a glycosaminoglycan containing a pentasaccharide that binds to and enhances the activity of antithrombin III by inducing a conformational change, thereby inhibiting thrombin and several activated coagulation factors (XIIa, IXa, XIa, and Xa) [23]. Heparin is not readily absorbed by the gastrointestinal tract and is thus administered either intravenously or subcutaneously. Heparins are comprised of UFH and LMWH. UFH consists of a mixture of polysaccharides with varying molecular weights averaging approximately 15,000 Daltons. LMWH has shorter polysaccharide chains and average molecular weights between 4000–6000 Daltons. LMWH does not inhibit thrombin as readily as UFH; however, LMWH and UFH are thought to inhibit factor Xa to a similar degree [24].

### 3.1. Unfractionated Heparin

UFH is the most pervasive anticoagulant used in the inpatient population worldwide for multiple reasons, including treatment or prophylaxis of VTE, stroke and transient ischemic attack (TIA), acute coronary syndromes, cardiac surgeries including cardioversion, and in the perioperative and critical care settings [5]. It is also used to flush lines to avoid contact factor activation such as in hemodialysis, ECMO, and cardiopulmonary bypass machines. In addition, UFH is considered safe for use in all populations including neonates, children, and pregnant women.

UFH has a short half-life of 30 min when administered as a continuous, intravenous (IV) infusion, and 90 min when administered subcutaneously via parenteral injections. It is easily reversible using protamine sulfate; however, in the absence of protamine, the short half-life allows for reversal by simple discontinuation of UFH administration [24]. Nevertheless, there are significant limitations to the use of UFH, as it has a highly variable dose–response relationship and as such requires frequent monitoring to ensure therapeutic levels, is unable to be administered orally, and is associated with complications such as heparin-induced thrombocytopenia (HIT) and increased risk of bleeding events as compared to LMWH [25].

Therapeutic dosing of UFH is typically achieved by the IV administration of an initial bolus followed by weight-based or calculated, fixed-dose heparin dosing via continuous infusion, that can be modified as needed depending on the bleeding risk [26,27]. Prophylactic UFH is typically administered in 5000 U subcutaneous parenteral injections, either two or three times per day; while there is conflicting evidence available on which regimen is more effective. Meta-analytic data suggests that 5000 U three times per day is more efficacious than twice per day for VTE prophylaxis, despite the higher bleeding risk [28]. After administration, UFH is removed from circulation via a combination of the saturable mechanism, by which heparin binds with high affinity to endothelial cells and is cleared by the reticuloendothelial system and the non-saturable mechanism, i.e., renal excretion [29].

The historical gold standard for UFH monitoring has been the use of serial aPTT that are typically measured frequently (within 2 h of initiation of continuous IV infusion, and every 6 h thereafter). The therapeutic range is approximately 1.5–2.5 times the patient’s baseline aPTT [23,24,30]. The UFH dose may then be adjusted in relation to the aPTT, and monitoring may become less frequent as the target range is achieved and sustained. While this therapeutic aPTT range is widely recognized and utilized by clinicians, there is only limited evidence supporting this guideline. There are several complicating factors that can make aPTT monitoring difficult and unreliable; aPTT is sensitive to other variables such as coagulation factor consumption in the setting of bleeding or thrombosis, decreased synthesis of coagulation factors in the setting of liver dysfunction or disorders such as hemophilia and von Willebrand disease, and interferences such as the presence of a lupus anticoagulant, which would prolong the baseline aPTT [31,32]. In addition, there is known to be wide variability in the sensitivity of aPTT reagents, and individual laboratories are therefore recommended to define their own therapeutic aPTT ranges for safe and reliable heparin monitoring [33].

UFH may also be monitored via anti-factor Xa (anti-Xa) activity, and the question of whether this method is more efficacious than the use of aPTT remains controversial. UFH anti-Xa assays specifically measure the ability of heparin-bound antithrombin III to inhibit factor Xa [34]. Studies have shown that the use of anti-Xa is more efficient in achieving the target therapeutic range of UFH as compared to aPTT; however, this has not been shown to have an effect on clinical outcomes [35,36]. In addition, while both aPTT and anti-Xa can be performed on the same automated coagulation analyzers, anti-Xa is reportedly difficult to standardize and has lower precision than aPTT tests [34,37,38]. Furthermore, many smaller hospitals and laboratories do not offer anti-Xa as it is more specialized and expensive, and in general there is less knowledge among clinicians surrounding the utility and interpretation of anti-Xa results as compared to aPTT. Anti-Xa is also not feasible in patients with recent direct oral factor Xa inhibitor use, as these patients may have residual anti-Xa activity at the start of UFH therapy [27]. A new product known as DOAC-Stop**^®^** (Haematex Research, Hornsby, Australia) has been shown to mitigate the interference of DOACs with anti-Xa and other standard coagulation assays, though despite its potential clinical utility, DOAC-Stop**^®^** has only been evaluated in clinical trials and is not yet available for commercial use [39].

On the other hand, anti-Xa may mitigate some of the issues surrounding aPTT monitoring in certain patients for which aPTT is unreliable, such as those with lupus anticoagulant. In addition, the aPTT may also be falsely prolonged in the setting of elevated C-reactive protein (CRP), thereby causing discrepancies in aPTT and anti-Xa results in this patient population. Anti-Xa is therefore recommended for monitoring heparin therapy in patients with elevated CRP [40]. Interestingly, a 2012 study of 539 hospitalized adults receiving UFH with a total of 2,321 paired aPTT and anti-Xa values found that there may be some clinical utility in measuring both aPTT and anti-Xa, since patients with disproportionately prolonged aPTT values as compared to anti-Xa had worse clinical outcomes. The study concluded that the concurrent measurement of aPTT and anti-Xa could be useful in stratifying bleeding risk and assist in determining the appropriate dosing regimen [36].

There are some exceptions to the use of aPTT or anti-Xa for UFH monitoring, including interventional cardiology patients, who often receive very high doses of UFH intraoperatively, such as during cardiac catheterization, coronary artery bypass grafting (CABG), or left ventricular implant device (LVAD) implantation, as well as patients on ECMO. The activated clotting time (ACT) is a point-of-care test used for high-dose heparin monitoring and measures the inhibition of the contact and common pathway (factor X-Xa) activation. ACT is the preferred test in these settings due to the short time from sampling to results, size and portability (allowing for measurement at bedside and during transport), user-friendliness and ability to be performed by non-laboratory personnel, no need for a central laboratory, and quick confirmation of UFH reversal with protamine sulfate [41]. However, ACT is prone to interference by other anticoagulants, especially DOACs, which are common in patients undergoing interventional cardiac procedures. This can result in largely variable clotting times, thus challenging the standard therapeutic target of 300 s and resulting in the potential for under- or over-dosing of UFH [42]. Additional challenges to UFH monitoring include patients with heparin resistance or antithrombin III deficiency, for which both aPTT and anti-Xa (and the addition of antithrombin III concentrates in antithrombin III-deficient patients, if clinically indicated) are typically used [27].

### 3.2. Low Molecular Weight Heparin

There are multiple commercially available preparations of LMWH worldwide, including enoxaparin, dalteparin, tinzaparin, and nadroparin. Each variant of LMWH is chemically and pharmacologically distinct, including different ratios of factor Xa vs. thrombin inhibition, meaning that the clinical efficacy and safety of each drug varies as well [27]. Enoxaparin has the widest range of clinical indications due to the breadth of clinical data regarding safety and efficacy across many patient populations. As such, it is the most commonly marketed and prescribed LMWH [43]. However, there are few studies to date that have compared clinical outcomes in patients taking different LMWH products. LMWH is administered via subcutaneous parenteral injection and has similar clinical indications as UFH, including the treatment and prophylaxis of VTE (including during pregnancy), treatment of myocardial infarction and unstable angina, and prevention of clotting in extracorporeal circuits [44]. LMWH is also the recommended anticoagulant for the treatment of cancer-associated VTE [45].

Clinicians usually weigh the pros and cons of anticoagulation with LMWH as opposed to UFH in order to determine the best regimen for their patients. LMWH is often preferable over UFH for several reasons. Firstly, it is more readily absorbed and involves less endothelium and protein binding, which results in greater bioavailability. It also has a longer half-life of 4 h, allowing for injection only once or twice daily. LMWH has been shown to have better correlation between dosage and anticoagulant response, which allows for fixed-dose administration and less frequent monitoring, if at all [24,27]. In addition, LMWH carries a lower risk of complications such as HIT, bleeding, and is associated with a lower risk of osteoporosis as compared to UFH [27,46]. LMWH injections also lend themselves to both inpatient and outpatient use, though long-term use is often associated with bruising and the deterioration of injection sites, and overall lower patient satisfaction as compared to oral anticoagulants [44,47].

Other limitations of LMWH as compared to UFH include a delayed onset of action (up to 30 min as opposed to instantaneous functionality in the case of intravenous UFH bolus), and the longer half-life makes urgent reversal more difficult [27]. Protamine sulfate can be used for reversal in the absence of alternative solutions; however, it is known to be less effective at reversing anti-Xa activity than antithrombin activity [48]. Since LMWH is renally cleared, it has a prolonged half-life in patients with renal failure, which is associated with a higher risk of accumulation and subsequent bleeding complications [49].

Anti-Xa is the gold standard for monitoring LMWH therapy, as aPTT is not significantly affected. LMWH is thought to have lower requirements for monitoring in general as compared to UFH, primarily due to fixed-dose administration, the longer half-life, and improved bioavailability, as discussed previously. In addition, studies have shown that the anticoagulant effect and risk of bleeding are not consistently correlated with plasma anti-Xa levels, and that weight-adjusted LMWH dosing has proven to be safe and effective, thus alleviating the need for close monitoring [50]. Furthermore, despite the increase in use of DOACs and resultant increase in availability of anti-Xa testing, not all patients or clinics (especially in rural areas) have routine access to a laboratory that offers anti-Xa testing, and increased turnaround time is impractical when making dose adjustments [27]. There is also known to be substantial variability between specific anti-Xa assays, which is affected by both the specific reagent and analyzer used, in addition to the aPTT reagent used for correlation of the therapeutic range. In turn, there is concern that this lack of standardization could potentially negatively affect clinical outcomes [51,52,53].

In patients for whom LMWH monitoring is required, anti-Xa levels should be obtained at their peak 4 h post-administration [54]. Dose adjustments in patients with renal insufficiency are based on anti-Xa levels; anti-Xa based enoxaparin dose reduction in particular has been shown to reduce the risk of bleeding in these patients [46,55]. Some studies have shown that the area under the thrombin generation curve, known as the endogenous thrombin potential (ETP), may be useful in LMWH and UFH monitoring, since it measures the total amount of thrombin formed in vivo as opposed to the limited quantity of thrombin formed in traditional coagulation assays (e.g., aPTT) [56,57,58,59]. While this assay may have potential, both for heparin monitoring and for hemostatic evaluation as a whole, it is not currently standardized or validated for clinical use [60].

## 4. Fondaparinux

Fondaparinux is a synthetic anticoagulant derived from a pentasaccharide sequence that functions similarly to LMWH in that it inhibits factor Xa, but not thrombin. It is known as an indirect factor Xa inhibitor because it inhibits factor Xa by way of selectively and reversibly binding to antithrombin III with a higher affinity than UFH and LMWH [61]. Fondaparinux is indicated in the treatment and prophylaxis of VTE, is often prescribed in the setting of acute coronary syndrome, and as an alternative to heparin in patients diagnosed with HIT, as it does not interact with platelets or platelet factor 4 [62]. It has 100% bioavailability after subcutaneous administration, reaching its peak concentration in 1.5–2 h after injection [63]. The half-life of fondaparinux is approximately 15–17 h, and anticoagulant functionality remains for 2–5 days after injection in patients with normal renal function. It is administered once daily and dosed according to body weight and indication for use (i.e., therapeutic vs. prophylactic dosing). Protamine sulfate is ineffective in the reversal of fondaparinux, and no specific reversal agents are directly approved for this purpose. However, the administration of recombinant activated factor VII has shown to aid in the cessation of bleeding in fondaparinux-related hemorrhage [64].

Fondaparinux does not require monitoring in the majority of cases; however, anti-Xa may be used in certain cases in which levels must be acutely determined, such as in high-risk patients with renal insufficiency, and should be performed approximately three hours after administration [62]. Since anti-Xa is not officially approved for fondaparinux monitoring, the therapeutic range is not well-established or standardized, and thus varies between clinical laboratories [65].

## 5. Parenteral Direct Thrombin Inhibitors

Beginning in 2000, several parenteral DTI (administered either intravenously or subcutaneously) were approved for use in the inpatient population. These include the synthetic r-hirudin analog bivalirudinas well as argatroban, which is a synthetic thrombin inhibitor derived from arginine. DTIs function by reversibly binding to the active sites of thrombin [66]. Intravenous DTIs are often used as alternative anticoagulants to UFH in patients with HIT; argatroban in particular has been found to significantly reduce thromboembolic complications in HIT patients [67]. Bivalirudin is frequently used in critically ill patients as an alternative to UFH. Both bivalirudin and argatroban are clinically indicated for thromboprophylaxis in patients undergoing percutaneous coronary intervention (PCI) when UFH is contraindicated. In addition, bivalirudin may be used in patients with unstable angina, acute coronary syndromes, and non-ST-segment myocardial infarction (NSTEMI) [5]. As in the case of all anticoagulants, hemorrhage is a risk of IV DTI therapy. No specific reversal agent is available for IV DTIs, though emergent administration of recombinant activated factor VII (FVIIa) has been reported to be beneficial in the treatment of severe bleeding [68].

The plasma half-lives of DTIs range from approximately 25 to 120 min, depending on whether they are administered via IV or subcutaneous injection [69]. Bivalirudin has an immediate onset of action, becoming therapeutic within 5 min of the start of infusion (according to therapeutic ACT values), and is approximately 20% renally excreted, with the rest cleared via proteolytic cleavage and hepatic metabolism [70]. Argatroban has peak plasma concentrations at approximately 10 h after the initiation of therapy, and is metabolized hepatically; as such, dose reduction is necessary in patients with liver dysfunction [69]. The most common tests for bivalirudin and argatroban anticoagulation monitoring are aPTT and ACT; however, thrombin time (TT), dilute thrombin time (dTT), chromogenic anti-IIa, and ecarin clotting time (ECT) have also been reported to be used. For aPTT monitoring in patients with HIT, the target aPTT ranges for bivalirudin and argatroban are 1.5–3.0 and 1.5–2.5 times the baseline aPTT, respectively [71].

## 6. Direct Oral Anticoagulants

Significant changes in anticoagulation practice have evolved in the past decade or so, following the development of DOAC medications. DOACs consist of direct activated factor Xa inhibitors and direct activated factor II inhibitors (also known as DTIs). Factors X and II are key proteins within the coagulation cascade. Factor Xa converts prothrombin to thrombin, or factor IIa, which converts soluble fibrinogen to fibrin, activates factors V, VIII, XI and XIII, and stimulates platelets. The four DOACs currently approved in the USA and Europe include rivaroxaban, apixaban, edoxaban, which are factor Xa inhibitors, and dabigatran, which is a DTI [72,73]. An additional factor Xa inhibitor, betrixaban, has been approved in the USA only [73,74]. Factor Xa inhibitors are aptly named, as they bind to the active site of factor Xa, which directly inhibits both free circulating and clot-associated factor Xa [4]. Factor IIa inhibitors such as dabigatran do not require a cofactor and act by directly, selectively, and reversibly binding to the catalytic site of thrombin [6,75].

In general, DOACs are indicated for thromboprophylaxis following major orthopedic surgery, treatment of VTE, and prevention of stroke in patients with non-valvular atrial fibrillation (NVAF). Rivaroxaban and dabigatran were initially approved in the Europe Union (EU) in 2008 for the prevention of VTE after hip or knee replacement surgery, followed by apixaban in 2011. Shortly after, all three agents were approved in the EU for prevention of stroke and systemic embolism in adult patients with NVAF [72]. In 2010 in the US, dabigatran was the first DOAC to be approved for stroke prevention in patients with NVAF followed by rivaroxaban and apixaban within two years. By 2014, all three drugs were available in the US for VTE prophylaxis and treatment. Edoxaban was then approved in 2015 for stroke prophylaxis in patients with atrial fibrillation and treatment of VTE. Three years later, rivaroxaban was approved for prevention of atherothrombotic events in patients with chronic coronary artery disease (CAD) or peripheral artery disease (PAD). Apixaban was also approved for the treatment of heparin-induced thrombocytopenia [73].

**Figure 2:**
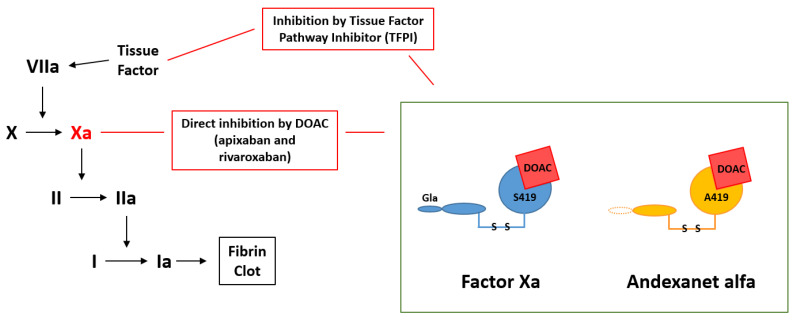
Reversal of DOACs by andexanet alfa. Abbreviations used—VIIa: Factor VIIa; X, Xa: Factor X, Xa; II, IIa: Factor II, IIa; I, Ia: Factor I, Ia; DOAC: Direct oral anticoagulant; TFPI: Tissue factor pathway inhibitor; S419: Catalytic domain, active binding site with serine: Gla: membrane-binding domain N-terminal γcarboxyglutamic acid; A419: Replacement of catalytic domain, active binding site with alanine.

DOACs are considered at least as effective as warfarin for anticoagulation and are associated with a lower incidence of intracranial hemorrhage [72,76]. Their benefits over warfarin include a reduced need for regular monitoring due to rapid onset, short half-lives, administration at a fixed dose, and fewer drug and dietary interactions. As they are short-acting, some require twice daily dosing. There was no specific reversal agent for a DOAC until 2015 when the FDA approved idarucizumab (Praxbind^®^, Boehringer Ingelheim, Ingelheim am Rhein, Germany) for reversal of dabigatran; in 2018 andexanet alfa (Andexxa^®^, Portola, South San Francisco, CA, USA) was approved for the reversal of apixaban and rivaroxaban [77]. Andexanet alfa is thought to have good clinical safety and efficacy, and has been found to reverse apixaban and rivaroxaban within minutes of administration (Figure 2) [78].

### 6.1. DOAC Monitoring

The general consensus among clinicians is that patients receiving DOAC therapy do not require routine monitoring or dose adjustment [79]. There are times when monitoring is beneficial, however, as in patients with severe bleeding, for detection of residual anticoagulant drug effects prior to surgery, before thrombolysis in acute stroke patients, following rescue from overdose, in the treatment in patients with extremes of body weight, to assess drug interactions, in cases of renal impairment, and in cases of suspected non-compliance [80].

The two tests that can help guide management for patients receiving DOACs include a screening assay for determination of the presence or absence of the anticoagulant (e.g., prior to administering thrombolytics or urgent/emergent surgery), and a quantification assay for detection of abnormal absorption/clearance/metabolism (e.g., extreme body habitus, renal insufficiency, medication interaction) [81]. Standard coagulation assays such as PT and aPTT can be used as first-line tests to provide a qualitative assessment of rivaroxaban and dabigatran, respectively, depending on the sensitivity of the PT and aPTT reagents [11,82]. In general, DTI tend to disproportionately prolong the aPTT rather than PT, while direct factor Xa inhibitors prolong the PT to a greater extent than the aPTT [5]. However, because of their limited sensitivities, PT and aPTT are not suitable for quantification of the anticoagulant effect. In general, there is a poor correlation between plasma concentrations of DOACs and PT/PTT prolongation. Nevertheless, a normal aPTT and/or PT cannot rule out the DOAC effect [83]. Thus, as indicated by the International Society for Laboratory Hematology (ISLH), the aPTT and PT are unpredictable in assessing DOAC activity [84].

Mass spectrometry, when calibrated with each drug individually, to be measured, is considered the gold-standard method for quantification of DOAC level and demonstrates good accuracy and precision over a broad concentration range. However, this test is very involved, time consuming, and not widely available. More rapid methods including dTT, ecarin methods, and chromogenic anti-Xa assays are potentially suitable means to measure DOACs, but must employ calibrators and controls specific for (or referenced against) the DOAC being measured [85]. Quantification of dabigatran is best achieved with dTT, ECT, or chromogenic anti-IIa assay. The European Heart Rhythm Association (EHRA) Practical Guide [86] and International Council for Standardization in Haematology (ICSH) recommendations [87] both suggest that a normal TT value excludes the presence of even low levels of dabigatran. However, TT is not suited for the quantitative assessment of dabigatran plasma concentrations in the expected clinical range [88]. In contrast, both dTT and ECT display a direct linear relationship with dabigatran concentration, and are thus suitable for quantitative assessment [87]. The ECT assay provides a direct measure of dabigatran activity, but it is not routinely available in some countries [89].

The anti-Xa assay can be used to measure DOAC (factor Xa inhibitor) activity via chromogenic reagents and a special DOAC calibrator [69]. Several studies have demonstrated the accuracy and sensitivity of drug-specific anti-Xa chromogenic assays for the quantitative measurement of rivaroxaban [90]. These assays (calibrated with rivaroxaban) can measure a wide range of rivaroxaban plasma concentrations that cover the expected levels after therapeutic doses. Similarly, the general recommendation for the assessment of apixaban exposure is anti-Xa chromogenic assays using specific apixaban standard calibrators. Global coagulation assays such as viscoelastic tests (ROTEM^®^ and TEG^®^) and thrombin generation assays have also been suggested as potential tests for the assessment of the anticoagulant effect of DOACs, though to date this has been assessed primarily in research [89].

There is no broad consensus on whether DOACs require monitoring, and if so, the best practices for doing so. Specific guidelines for DOAC therapy monitoring as well as therapeutic ranges, which vary significantly between patient populations and for each DOAC, are still limited in the literature. This is also dependent on the ability to standardize the wide range of available tests, as well as inter-reagent variability. Future clinical trials may provide better guidelines for DOAC monitoring practices across various patient populations [82].

### 6.2. DOAC Clinical Studies

Recent studies have investigated the use of DOACs combined with a P2Y12 inhibitor as compared to warfarin in patients with nonvalvular atrial fibrillation undergoing PCI (PIONEER AF-PCI for rivaroxaban, RE-DUAL PCI for dabigatran, AUGUSTUS for abixaban, ENTRUST-AF PCI for edoxban), and have shown reduced risk of bleeding without increased risk of thrombosis [73]. The RE-ALIGN trial looked at dabigatran as compared to warfarin in patients with mechanical heart valves and established that DOACs are contraindicated in patients with mechanical valves [91]. Despite the results of that study, there is a growing interest to investigate use of DOACs in the setting of bioprosthetic valves. Another important cardiovascular event in which DOACs may be able to play a role is stable atherosclerotic cardiovascular disease [92]. As previously mentioned, rivaroxaban is already approved in the US for prevention of cardiovascular events in patients with chronic CAD or PAD, its efficacy having been demonstrated in the COMPASS study in 2018 [93].

## 7. Discussion

In the past two to three decades, there has been a shift from the ubiquitous prescription of heparin (specifically UFH) and VKA in favor of newer forms of anticoagulation. However, certain clinical scenarios pose challenges associated with the management of anticoagulation. These include different variables in the inpatient versus outpatient settings, short versus long-term anticoagulation, dosage and dose adjustment methods, determining whether anticoagulation monitoring is necessary, and if so, what the appropriate assays are and how to interpret them. Throughout decades of management with heparin and VKA, common screening coagulation tests such as aPTT, PT, and INR have become staples of clinical practice. Along with the development of newer agents including the various forms of LMWH, DTIs, and factor Xa inhibitors, came focused clinical and translational research fine-tuning the pharmacophysiology and creating new practice guidelines specifying the utility of each anticoagulant, the interaction of multiple concomitant anticoagulants (as well as with other non-anticoagulant medications), and stratifying the risks and benefits of each.

Worldwide, VKAs including warfarin, acenocoumarol, and phenprocoumon remain the most commonly prescribed oral anticoagulants—until recently, they were also the only option available—most likely because they are inexpensive, accessible, easily monitored, adjustable, and reversible if needed. However, DOACs are becoming increasingly prevalent, especially with their approval in Europe and the US for thromboprophylaxis and stroke prevention in patients with atrial fibrillation, a spot previously held only by warfarin [94,95]. The more stable and predictable anticoagulant effects of DOACs, as seen in fewer food, drug, and supplement interactions, lend themselves to increased patient and physician satisfaction alike, especially in patients who require long-term anticoagulation [96]. While DOACs are still accompanied by risks of thromboembolic and hemorrhagic complications, when used properly they rarely require dose adjustments and have a broad therapeutic index. In patients with normal renal function who are deemed good therapeutic candidates, and for whom anticoagulation monitoring is not needed, DOACs seem a logical choice [97].

Likewise, the increased use of LMWH and parenteral DTIs in the inpatient population in recent years shows an overall trend away from anticoagulants that require frequent monitoring, such as UFH. However, in the critical care and surgical settings, the ability to adequately monitor anticoagulation therapy can be critical, as the dynamic clinical status of patients often results in the trend toward a hyper- or hypocoagulable state, and resultant imbalances in anticoagulation can increase the risk of thrombosis or hemorrhage. In addition, many of the newer anticoagulant agents do not have specific reversal agents, and in tenuous clinical scenarios, the ability to quickly and accurately determine the extent to which the patient is anticoagulated is invaluable. This especially applies to patients with renal and/or hepatic function, for whom the accumulation of anticoagulation as a result of impaired excretion poses heightened risks of severe adverse effects. While there are tests available for factor Xa inhibitor and DTI monitoring, there lacks broad standardization and calibration of assays, as well as concrete, evidence-based guidelines [98]. Furthermore, many of these assays are highly specialized and expensive (i.e., mass spectrometry and chromogenic factor assays), and are thus not feasible in centers without sophisticated laboratory testing. Despite significant advances in hemostasis and thrombosis research in recent decades, it seems that targeted anticoagulant reversal agents and monitoring practices are developed more slowly than the anticoagulants themselves. We anticipate significant advancements in the practices surrounding anticoagulant monitoring and reversal in the near future that will improve clinical outcomes.

## Figures and Tables

**Table 1:** Anticoagulants categorized by mechanism of action.

Anticoagulation Category	Medication Name(s)	Mechanism of Action	Route(s) of Administration
Vitamin K Antagonists	Warfarin, Acenocoumarol, Phenprocoumon	Inhibition of vitamin K epoxy reductase to decrease the synthesis of vitamin K-dependent coagulation factors	Oral
Heparin (Unfractionated)	Heparin	Inhibition of thrombin and several activated coagulation factors (including Xa) by binding to and enhancing the activity of antithrombin III	Intravenous or Subcutaneous paretneral injection
Heparin (Low Molecular Weight)	Enoxaparin, Dalteparin, Tinxaparin, Nadroparin	Binds to antithrombin III and inhibits thrombin to a much lesser extent than unfractionated heparin; primarily inhibits factor Xa	Subcutaneous parenteral injection
Factor Xa Inhibitors	Fondaparinux *, Rivaroxaban, Apixaban, Edoxaban, Betrixaban	Prevents the cleaving of prothrombin by factor Xa to form thrombin	Fondaparinux- Subcutaneous parenteral injection Rivaroxaban, apixban, edoxaban, betrixaban- Oral
Factor IIa Inhibitors (Direct Thrombin Inhibitors)	Dabigatran, Bivalirudin, Argatroban	Directly binds to and inhibit thrombin	Dabigatran- Oral Bivalirudin- Intravenous Argatroban- Intravenous or Subcutaneous parenteral injection

* Fondaparinux, while technically a synthetic low molecular weight heparin, is considered an indirect factor Xa inhibitor.

## Data Availability

No new data were created or analyzed in this study. Data sharing is not applicable to this article.
